# Oral care tablet containing kiwifruit powder affects tongue coating microbiome

**DOI:** 10.1002/cre2.591

**Published:** 2022-05-17

**Authors:** Makoto Fukui, Hiroki Asakuma, Hiroko Horiuchi, Hiroshi Takii, Masami Yoshioka, Daisuke Hinode

**Affiliations:** ^1^ Department of Hygiene and Oral Health Science Tokushima University Graduate School of Biomedical Sciences Tokushima Japan; ^2^ Applied Research Laboratory Ezaki Glico Co., Ltd. Osaka Japan; ^3^ Research Promotion and Management Office, Ezaki Glico Co., Ltd. Osaka Japan; ^4^ Faculty of Health and Welfare Tokushima Bunri University Tokushima Japan

**Keywords:** kiwifruit powder, microbiome, oral care tablet, tongue coating

## Abstract

**Objectives:**

Tongue coating, a kind of biofilm formed on the tongue dorsum, is the cause of various clinical conditions, such as oral halitosis and periodontal diseases, because *Fusobacterium nucleatum* acts as a bridge between other oral bacteria and periodontopathogenic bacteria in biofilm formation. Our previous clinical study revealed that taking oral care tablets containing kiwifruit powder significantly reduced not only tongue‐coating index and volatile sulfur compounds but also total bacteria and *F. nucleatum* in tongue coating. In this study, we analyzed the microbiome of tongue coating samples obtained before and after oral care tablets intake to clarify whether this tablet is a useful tool for daily tongue care.

**Methods:**

Thirty‐two healthy young adults were enrolled, and a crossover clinical trial was conducted. We instructed subjects to remove tongue coating by tongue brush for intervention I, to keep the oral care tablet containing kiwifruit powder on the tongue dorsum and to let it dissolve naturally for intervention II. Microbial DNA was isolated from the collected tongue coating samples in each subject, then 16S rRNA next‐generation sequencing, operational taxonomic unit clustering, and statistical analysis were performed.

**Results:**

The microbiome analysis revealed that the oral care tablet in intervention II prompted a significant change in the tongue microbiota composition, a significant reduction in the relative abundance of *Prevotella* and *Porphyromonas*, and an increase in Firmicutes/Bacteroidetes ratio when compared to that in intervention I.

**Conclusion:**

These results suggested that the oral care tablet might contribute to the improvement of the oral condition due to its good influence on the tongue coating microbiome.

## 1 INTRODUCTION

From ancient times to the present, oral malodor has been a serious problem for people because it may interfere with their daily activities and interpersonal relations. Oral malodor is caused mainly by volatile sulfur compounds (VSCs) in mouth air, and these include hydrogen sulfide (H_2_S), methyl mercaptan (CH_3_SH), and dimethyl sulfide [(CH_3_)_2_S] (Tonzetich, 1971). Oral periodontopathogenic bacteria are capable of producing large amounts of VSCs (Ayers & Colquhoun, 1998; Nakano et al., 2002; Shibuya, 2001). It has also been reported that approximately 60% of VSCs originate from tongue coating in patients with periodontitis (Yaegaki & Sanada, 1992). Tongue coating is a kind of biofilm formed on the tongue dorsum and consists of epithelial cell debris, blood cells, and food debris in addition to oral bacteria that metabolize these substrates.

*Fusobacterium nucleatum* is a key bacterium among a number of oral bacteria on tongue coating. *F. nucleatum*, a gram‐negative anaerobic oral bacterium, produces large amounts of VSCs, including H_2_S and CH_3_SH (Claesson et al., 1990), and is a representative of the occurrence of oral malodor. *F. nucleatum* also plays a critical role in oral biofilm architecture by acting as a bridge between early Gram‐positive and late Gram‐negative colonizers (Kolenbrander et al., 2010). In other words, *F. nucleatum* has a central role in the biofilm formation, VSCs production, and pathogenesis of oral diseases on the surface of the tongue and tooth.

Therefore, it is considered that the removal of tongue coating including *F. nucleatum* is effective to improve oral malodor and oral condition. However, we had previously reported that 70% of subjects with highly accumulated tongue coating did not recognize their tongue coating and that half of the subjects had no habit of daily tongue cleaning (Amou et al., 2014). It will be necessary to prove the positive effects of removing tongue coating and propose easy tongue coating care that can be made a habit to prevent halitosis and improve the oral condition.

We focused on an oral care tablet that can easily remove tongue coating. Previously, in a crossover clinical trial in healthy young subjects who took oral care tablets or used tongue brush, we reported that oral care tablets significantly reduced Winkel tongue‐coating index, VSCs, total bacteria, and *F. nucleatum* in tongue coating (Matsumura et al., 2020). As explained above, *F. nucleatum* has a central role in biofilm formation and is associated with dysbiosis of the oral microbiome and the pathogenesis of oral diseases. According to the result that *F. nucleatum* in tongue coating was reduced by taking oral care tablets in our previous clinical trial, it is presumed that oral care tablets cause a change in the tongue microbiome and has positive effects on the oral condition. Previous clinical studies did not report in detail the composition of the bacterial flora in each sample and could not verify the usefulness of its chemical control against biofilm. Therefore, to assess the effect of oral care tablets on the tongue bacterial flora, we analyzed the microbiome of tongue coating samples obtained in the clinical trial using 16S rRNA next‐generation sequencing.

## 2 MATERIAL AND METHODS

### 2.1 Subjects

As reported previously (Matsumura et al., 2020), the sample size was obtained as follows: the data of the number of oral bacteria after using the oral care tablet and that of tongue brush was obtained from the results of five participants in our preliminary study. The primary variable was that the number of bacteria (Log [cells/ml]) and the sample size was based on a two‐tailed *t* test with a significant difference level of 0.05, a power level of 0.90, and an anticipated effect size *d* = difference of means/standard deviation = 1.19. The required sample size was 16 in each group for a total of 32. Thirty‐two healthy students (5 males and 27 females; mean age 21.5 ± 2.1 years), who belonged to Tokushima University were enrolled in this study (Matsumura et al., 2020). Before enrollment, the subjects were informed about the methods and objectives of the study, and they provided written informed consent. Participants were dentulous men and women, 18 years of age or older. Current smokers, pregnant women, and participants who had received antibiotic treatment within the previous 2 weeks or who showed allergy against kiwifruit obtained from the preliminary survey regarding the fruit allergies with medical history form were excluded from the study.

### 2.2 Study design

**Figure 1:**
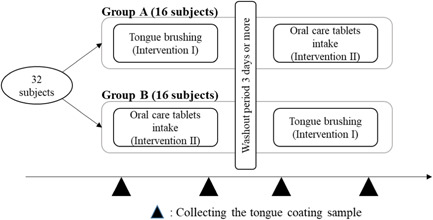
Outline of the crossover study.

The crossover clinical trial was conducted between Group A (16 subjects) and Group B (16 subjects) as previously reported (Matsumura et al., 2020). Participants were divided into two groups by considering only the male‐female ratio. Group A performed in the order of tongue brushing (Intervention I) and oral care tablet intake (Intervention II), whereas Group B performed in the order of Intervention II, Intervention I (Figure 1). These crossover studies had a washout period of 3 days or more. Before the intervention, each subject was asked to refrain from eating, drinking, and tooth brushing during the periods from waking up to the end of the trial and tongue cleaning within the past 3 days. Each tongue coating sample was collected before and an hour after the intervention using a sterile 5‐mm‐diameter cotton stick by swabbing the tongue dorsum three times from back to front (approx. 2‐cm‐long swabbing motions). Samples were suspended in 5 ml of distilled water and dispensed into vials. Collected samples were stored at −80°C until used for DNA preparation.

Interventions were performed as reported previously (Matsumura et al., 2020). For tongue brushing (Intervention I), we instructed subjects about tongue cleaning by scrubbing 10 times from back to front with a tongue brush (Supporting Information: Figure 1a) and then washing with 10 ml water. The subjects repeated the above procedure two times. Regarding the protocol of oral care tablet intake (Intervention II), we instructed subjects to take an oral care tablet (Supporting Information: Figure 1b) and to keep it on the tongue dorsum to let it dissolve naturally. Each participant digested two tablets. Two tablet‐intake was effective for the prevention of tongue coating deposition (Yoshimatsu et al., 2006); therefore, we decided on the 2‐tablet intake in this study. The oral care tablet was provided by Ezaki Glico Co., Ltd. (Osaka, Japan). Supporting Information: Table 1 shows the composition of the tablet.

### 2.3 DNA extraction and sequencing of 16S rRNA gene amplicons

The tongue coating microbiome in 31 subjects was analyzed except for one subject whose microbial DNA concentration extracted from the tongue coating sample was low and DNA amplification using polymerase chain reaction was not observed. Microbial DNA was isolated from the collected samples using the ISOSPIN Fecal DNA kit (Nippon Gene, Toyama, Japan). Bead‐beating was performed using the FastPrep‐24 instrument (MP Biomedicals, Solon, OH, USA) at a speed of 6 m/s with a bead‐beating time of 3 × 60 s as described previously (Tourlousse et al., 2021). The V1‐V2 variable region (27Fmod‐338R) was sequenced on an Illumina MiSeq (Illumina, San Diego, CA, USA) (Kim et al., 2013). The 16S rRNA V1–V2 amplicon was amplified using universal bacterial 16S rRNA gene primers: forward primer TCGTCGGCAGCGTCAGATGTGTATAAGAGACAGAGRGTTTGATYMTGGCTCAG and reverse primer GTCTCGTGGGCTCGGAGATGTGTATAAGAGACAGTGCTGCCTCCCGTAGGAGT. We attached dual indexes and Illumina sequencing adapters to polymerase chain reaction products using the Nextera XT Index Kit (Illumina). After purification of the amplicon, mixed samples were prepared by pooling approximately equal amounts of polymerase chain reaction amplicons from each sample. A sample library was sequenced using MiSeq Reagent Kit V3 (2 × 300 bp) and a MiSeq sequencer, according to the manufacturer's instructions.

### 2.4 Analysis pipeline for 16S rRNA data

Sequences were analyzed as described previously (Said et al., 2014). Based on sample‐specific barcodes, reads were assigned to each sample followed by the removal of reads lacking both forward and reverse primer sequences. Data were further denoised by the removal of reads with average quality values <25. Chimeric sequences were detected and removed by following both the reference (Greengenes ver. 12.10) and de‐novo based approaches using USEARCH/UCHIME 6.1. Finally, filter‐passed reads were used for further analysis after trimming off both primer sequences.

### 2.5 Operational taxonomic unit (OTU) clustering

From the filter‐passed reads, 5000 high‐quality reads/samples were randomly chosen. The total reads were then sorted according to average quality value and grouped into OTUs using UCLUST (http://www.drive5.com/) with a sequence identity threshold of 96%. Taxonomic assignments of each OTU were made by similarity searching against the publically available 16 S (RDP ver. 10.27 and CORE update September 2, 2012) and the NCBI genome database using the GLSEARCH program.

### 2.6 Statistical analysis

All statistical analyses were conducted using the open‐source software program R, Version 3.6.2 (https://cran.r-project.org/). After confirming the normality or not by statistical analysis, we chose a parametric test or a nonparametric test based on the results. Statistical evaluation of two groups was performed using the Wilcoxon signed‐rank test, Mann–Whitney's *U* test, or the paired *t* test. For beta diversity comparisons, Bray–Curtis dissimilarities were calculated and compared with the R package vegan (Oksanen et al., 2018). Statistical evaluation of beta diversity was assessed using the Mann–Whitney's *U* test. The difference in the microbial community composition (beta diversity) was tested using permutational multivariate analysis of variance (PERMANOVA) through the vegan R package command adonis with permutations set to 1000. A value of *p* < .05 was considered statistically significant.

### 2.7 Ethics

The Ethics Committee of Tokushima University Hospital approved this study (protocol approval number 2923). The method and objectives of this study were explained to the subjects, who provided written informed consent before their participation in the study.

## 3 RESULTS

**Figure 2:**
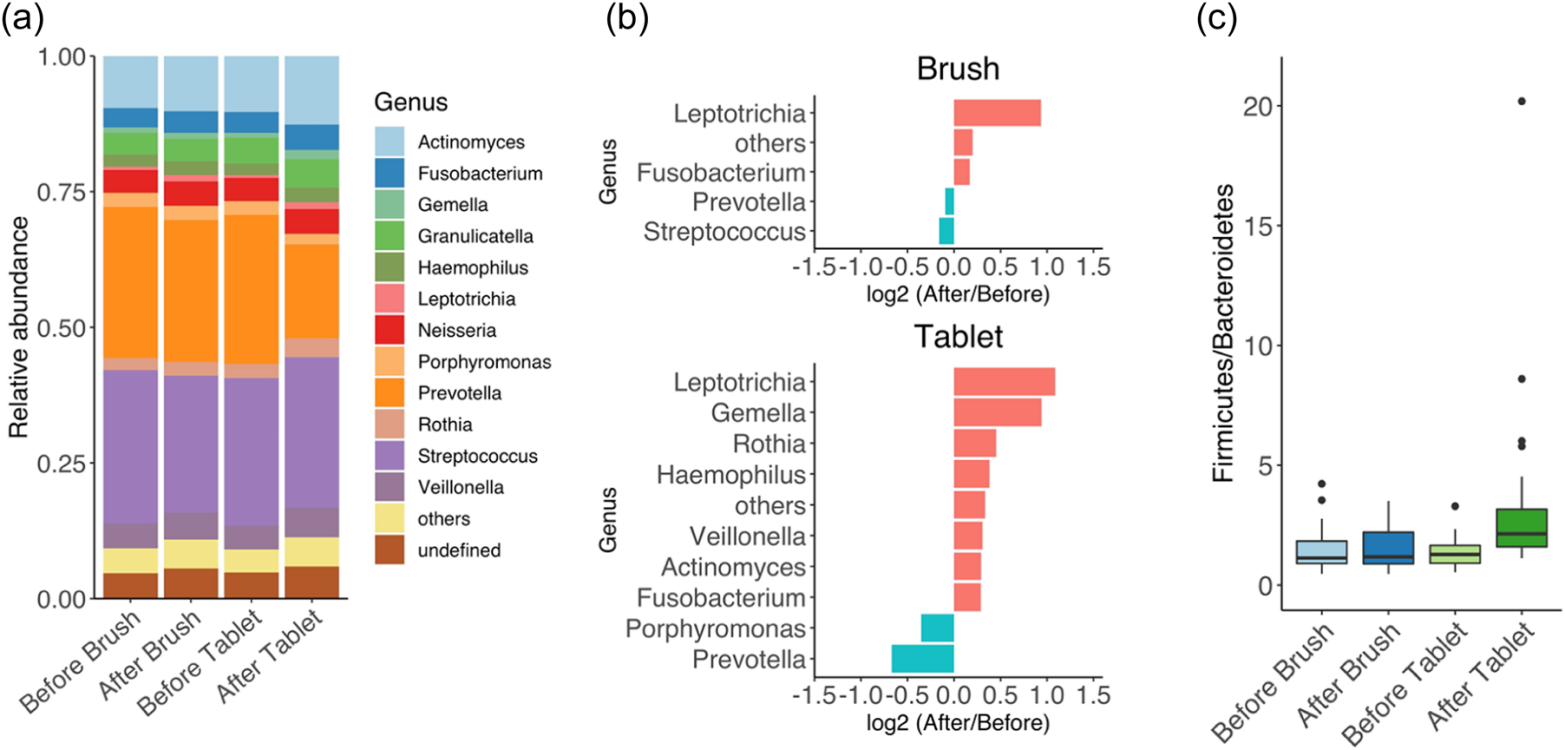
The bacterial composition of tongue coating samples before and after an intervention. (a) Relative abundance of major taxa (genus) groups. (b) The genus significantly changed after intervention in brush and tablet groups. Positive log2 fold change represents bacterial genera increased in “After” relative to “Before.” Negative log2 fold change represents bacterial genera increased in “Before” relative to “After.” *p* < .05 (Wilcoxon's signed‐rank test). (c) The Bacteroidetes/Firmicutes ratio.

Figure 2a shows the relative abundance of major taxa (genus) groups. The genera with a relative abundance of less than 5% in all samples collected from all subjects were summarized as “others.” Figure 2b shows the genus that significantly changed between before and after the intervention. The microbiome analysis revealed that more genera significantly changed after treatment in Tablet groups (Intervention II) than that in Brush groups (Intervention I). In particular, tongue brush significantly lowered the relative abundance of *Streptococcus* and *Prevotella*, while oral care tablets *Prevotella* and *Porphyromonas*. The number of *Fusobacterium* in oral care tablets in Figure 2b seems to appear larger; however, no significant difference in the *Fusobacterium* was observed between the tongue brush group and oral care tablet group in Figure 2b (Mann–Whitney's *U* test: *p*‐value = .63).

The Firmicutes/Bacteroidetes (F/B) ratio was calculated (Figure 2c). The F/B ratio in Brush groups had no change between before and after the intervention. On the other hand, the ratio in Tablet groups tended to increase after the intervention.

**Figure 3:**
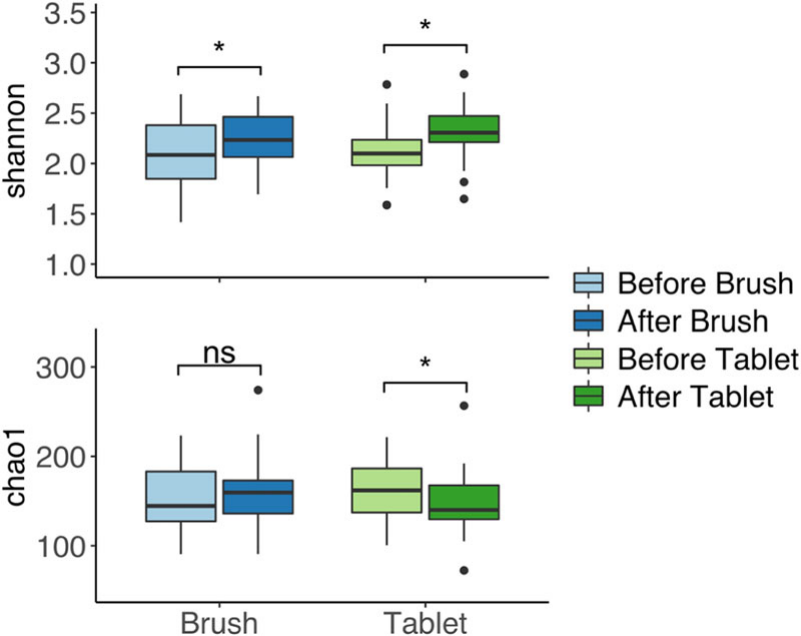
Changes in the alpha diversity index (Shannon index and Chao1 index) in brush groups in experimental I and tablet groups in experimental II. ns, not significant. *p < .05 (paired *t* test).

**Figure 4:**
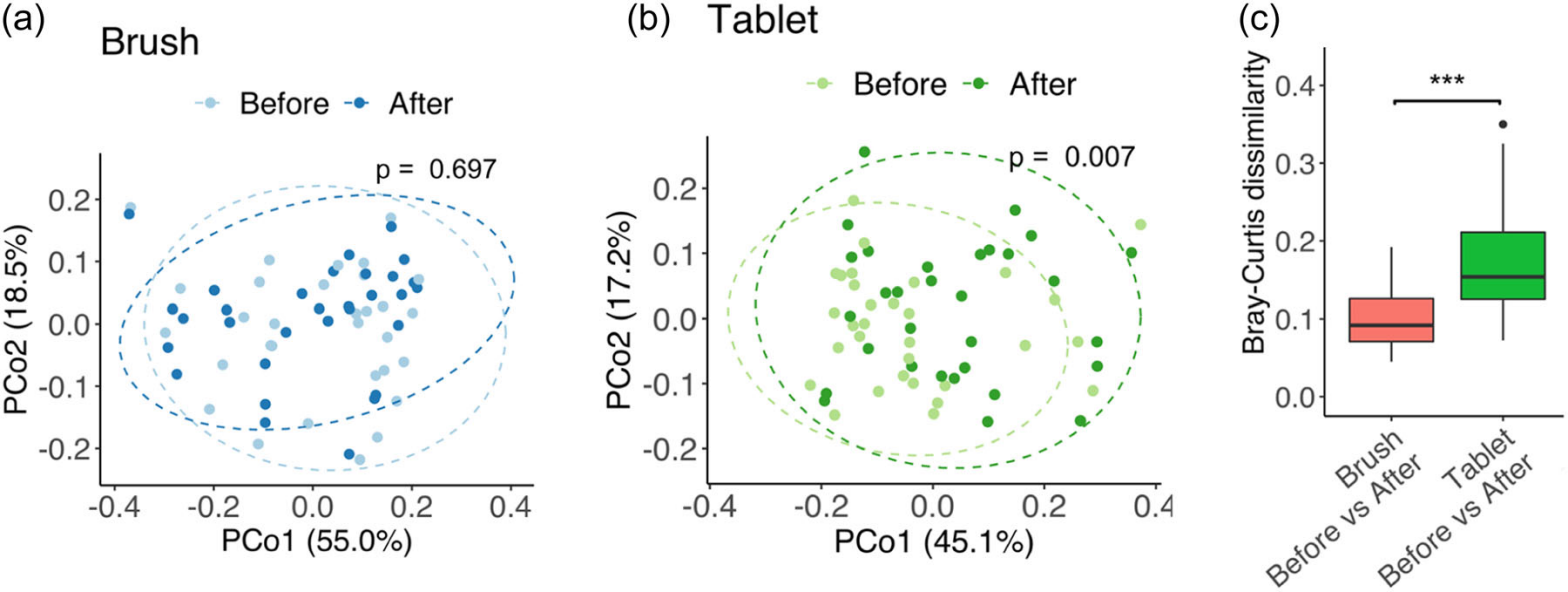
Principal coordinates analysis (PCoA) plot based on the Bray–Curtis dissimilarity showed a marked separation between before and after the intervention. (a) Brush and (b) tablet. The statistical significance among the two groups was determined with PERMANOVA. A value of *p* < .05 was considered statistically significant. (c) Box plots of Bray–Curtis dissimilarities between each sample. ***p < .001 (Mann–Whitney's *U* test)

Regarding alpha diversity, the Shannon index significantly increased in both interventions (Brush and Tablet), whereas the Chao1 index significantly decreased only in Tablet groups (Figure 3). No significant changes in OTUs were found in either group (data not shown). Regarding beta diversity visualized in the Principal Coordinates Analysis (PCoA) plot based on the Bray‐Curtis dissimilarity coefficient, there was no significant difference in Brush groups (Figure 4a), while the oral care tablet had a significant impact on the microbiota composition (Figure 4b). Figure 4c showed the box plots of Bray‐Curtis dissimilarities between samples (“Brush before vs. Brush After” and “Tablet before vs. Tablet After”). The analysis of Bray‐Curtis dissimilarity demonstrated that “Tablet Before vs. Tablet After” had a significantly higher dissimilarity than “Brush Before vs. Brush After.”

## 4 DISCUSSION

It was demonstrated that oral care tablets containing kiwifruit powder affected the bacteria in tongue coating from the result of the microbiome analysis in this clinical trial. In our previous research, we compared the effects of the oral care tablet and the tongue brush to clarify their potential to be effective tongue care (Matsumura et al., 2020). A tongue brush, which has generally been used for tongue care, has an effect on the removal of tongue coating (Amou et al., 2014). However, there are some problems with the tongue brush. Quirynen et al. (2004) reported that there might be a possibility of damaging the mucous membrane of the tongue by the brush bristle, and there was also a possibility of triggering the gag reflex during tongue brushing. Furthermore, the results of our previous clinical study revealed that a tongue brush is able to remove the tongue coating and reduce halitosis but has no effect on tongue coating bacteria (Matsumura et al., 2020). On the other hand, our results confirmed that oral care tablets significantly reduced total bacteria and *F. nucleatum* in tongue coating (Matsumura et al., 2020). This oral care tablet has a rough surface, which allows easy removal of the tongue coating while taking, and also contains several ingredients such as cysteine protease “actinidin” extracted from kiwifruit and some food acidulants (Supporting Information: Table 1). In brief, it is suggested that this oral care tablet has both physical and chemical action on bacteria in tongue coating (Mugita et al., 2016; Nohno et al., 2012). According to these previous results, we presumed the oral care tablet causes a change in the tongue microbiome. Hence, to assess the change of tongue bacterial flora due to oral care tablets or tongue brushes, we analyzed the microbiome of tongue coating samples obtained in the clinical trial.

The tongue dorsum is the largest surface in the mouth, and its papillary structure is complicated and highly colonized by bacteria (Gordon & Gibbons, 1966; Kojima, 1985; Nakano et al., 2002). The tongue surface could be an important reservoir for periodontal pathogens, which could cause various clinical conditions, such as oral halitosis and periodontal diseases (Faveri et al., 2006). *Fusobacterium nucleatum* is a key bacterium among a number of oral bacteria in the coating of the tongue dorsum. *F. nucleatum* has remarkable adherence properties, coaggregating with a wide array of microorganisms in the oral cavity through the expression of numerous adhesins (Brennan & Garrett, 2019). Due to these properties, *F. nucleatum* plays a role of a “bridge” between early Gram‐positive colonizers (e.g., *Streptococcus*) and late Gram‐negative colonizers, including periodontopathogenic bacteria (e.g., *Porphyromonas gingivalis*, *Prevotella intermedia*), and makes a great contribution to the biofilm maturation and pathogenicity in the oral cavity.

This microbiome analysis showed that the ratio of *Fusobacterium* tended to increase slightly in both interventions (Brush and Tablet) (Figure 2b). In contrast, the relative abundance of *Streptococcus* and *Prevotella* in Brush groups, and *Prevotella* and *Porphyromonas* in Tablet groups were significantly decreased due to intervention. In our previous study, we presented evidence that this oral care tablet reduced the number of total bacteria and *F. nucleatum* on the tongue (Matsumura et al., 2020). Therefore, these results indicate that the relative increase in the abundance of *Fusobacterium* might have been caused by the decrease of other bacterial abundances, such as *Prevotella* and *Porphyromonas*. In reference to the previous and this study, oral care tablets might produce effects on Gram‐negative bacteria, such as *Fusobacterium, Prevotella*, and *Porphyromonas*.

Tongue coating consists of epithelial cell debris, blood cells, food debris, and oral microorganisms, such as *P. gingivalis* and proteases of *P. gingivalis*, which are involved in the aggregation and adhesion to host cells (Morioka et al., 1992; Yoshioka et al., 1994). However, the ability of *P. gingivalis* to adhere to gingival fibroblasts decreases in an acidic environment (Yoshioka et al., 1994). It has been reported that actinidin, cysteine protease in tablets used in this study, reduces tongue coating in elderly subjects, and digest fimbriae to disrupt the biofilm structure and inhibit biofilm formation in vitro (Mugita & Nambu, 2017). Although the detailed mechanism against tongue microbiome by taking tablets remained unclear, it is possible that the effects of both organic acids and actinidin contained in the tablets disturbed the adherence of Gram‐negative bacteria to the tongue dorsum, then inhibited the aggregation and growth of bacteria and affected the structure of tongue coating.

This microbiome analysis yielded the result that the F/B ratio tended to increase after taking oral care tablets (Figure 2c). Firmicutes largely dominate the microbial communities in the oral cavity and include *Streptococcus* and *Veillonella*, whereas Bacteroidetes include several known periodontopathogenic bacteria, such as *P. gingivalis*, *P. intermedia*, and *Tannerella forsythia*. Moreover, analysis of Bray–Curtis dissimilarity showed that “Tablet Before vs. Tablet After” had a higher dissimilarity than “Brush Before vs. Brush After” (Figure 4c). According to these results, the oral care tablet yields a change in the tongue microbiome and weakens the integrity of biofilm. Thus, we considered that the oral care tablet might cause the reduction of pathogenicity in the oral cavity. Meanwhile, results that the tongue brush did not yield any significant change in the tongue microbiome were similar to previous studies (Laleman et al., 2018; Masago et al., 2020). These findings suggest that even physical removal of tongue coating would not suffice to suppress bacterial pathogens on the tongue, and the combined use of chemical actions including functional ingredients is useful even while tongue brushing. A 2‐tablet daily intake is expected to change the tongue coating microbiome.

It has been demonstrated that female sex hormones stimulated the growth of *P. intermedia* (Kornman & Loesche, 1982) and may thus contribute to the fact that the level of this periodontopathogen increases in periodontal sites of pregnant women (Kornman & Loesche, 1980). Focusing on the fact that the composition ratio of *Prevotella* was significantly reduced in this study, it might be useful for application to oral care in pregnant women during the morning sickness period when brushing is difficult. Periodontopathogenic bacteria, which also inhabit the tongue coating, are associated not only with oral malodor but also with periodontal disease and systemic diseases. For instance, tongue‐coating is a risk indicator of aspiration pneumonia in the edentate elderly (Abe et al., 2008). Periodontal disease caused by deterioration of oral health has been shown to be closely associated with diabetes (Demmer et al., 2008; Graziani et al., 2018) and Alzheimer's disease (Ide et al., 2016; Kamer et al., 2008; Noble et al., 2009). Furthermore, recent studies demonstrated that patients with colorectal cancer have identical *F. nucleatum* strains in their oral cavity and tumors (Komiya et al., 2019) and suggest that it may be associated with carcinogenesis and cancer progression (Abed et al., 2016; Brennan & Garrett, 2019). It has also been reported that *Porphyromonas* in tongue coating was increased in metabolic diseases (Li et al., 2021). In other words, in addition to improvement of the halitosis and oral condition, the oral care tablet might have a secondary effect on the prevention of systemic diseases.

On the other hand, there are still some questions in the research on the oral care tablet. As a first question, these results indicate that the change in the microbiome due to oral care tablets might be short‐term. Actually, in this clinical trial, we only evaluated the short‐term effect of the oral care tablet by the collection of tongue coating samples an hour after taking two tablets. Meanwhile, it was reported that a 1‐day usage of oral care tablets did not alter the tongue microbiomes of healthy subjects, who took three oral care tablets in a day and whose tongue coating sample was collected after waking up (Maruyama et al., 2020). Moreover, the analysis of the Human Microbiome Project data sets clarified that the oral habitat has the most stable microbiota in the human body (Zhou et al., 2013). Further research is needed to verify whether long‐term usage of oral care tablets yields the continuous improvement of the microbiome. As a second question, the subjects of this clinical trial were healthy young adults. To verify the preventive effect of the oral care tablet on oral diseases and systemic diseases, we need to conduct clinical tests on elderly people and subjects who are at risk of periodontal disease or systemic diseases. It has been reported recently (de Jesus et al., 2020) that sex might affect the normal microbial flora. This might be influenced by the oral treatment being used. The number of females was large in our study compared to that of males (male‐to‐female ratio = 5:27); therefore, different results using oral care tablets may be obtained if there was a research subject with different male‐female ratios. Further research is needed in consideration of gender differences. We will address these questions and clarify its potential as daily oral care in future studies.

In conclusion, this study revealed that the oral care tablet showed a significant change in the tongue microbiota composition, a significant reduction in the relative abundance of *Prevotella* and *Porphyromonas*, and an increase in the F/B ratio. In reference to the previous and present study, we suggest that this oral care tablet has positive effects not only on halitosis but also on oral conditions, and its effect was due to the action of the chemical control against biofilm. However, we only evaluated the short‐term effect of the tablet. Further studies are required to verify whether long‐term intake of oral care tablets will provide preventive benefits for oral and systemic diseases.

## 5 CONCLUSION

These findings suggested that the oral care tablet containing kiwifruit powder might contribute to the improvement of oral condition due to its good influence on the microbiome in tongue coating.

## AUTHOR CONTRIBUTIONS

The authors' contributions are as follows: Makoto Fukui, Masami Yoshioka, and Daisuke Hinode designed, coordinated, and performed the study and drafted the paper. Hiroki Asakuma, Hiroko Horiuchi, and Hiroshi Takii performed the experiments and drafted the paper. All authors reviewed the paper critically for content and approved it for submission.

## CONFLICTS OF INTEREST

Hiroki Asakuma, Hiroko Horiuchi, and Hiroshi Takii are employees of Ezaki Glico Co., Ltd., and part of this study was funded by Ezaki Glico Co., Ltd. The remaining authors declare no conflicts of interest.

## Supporting information

Supporting information.

Supporting information.

## Data Availability

The data that support the findings of this study are not shared because of the perspective of trade secrets regarding product manufacturing.
