# Supplementary figures and images for: Oral care tablet containing kiwifruit powder affects tongue coating microbiome
Source: Clin Exp Dent Res. 2022 May 17;8(3):721–8. doi: 10.1002/cre2.591 (PMC9209808; doi:10.1002/cre2.591)

　　　　　　　　　　　　　　　A　　　　　　　　　　　　　　　　　B


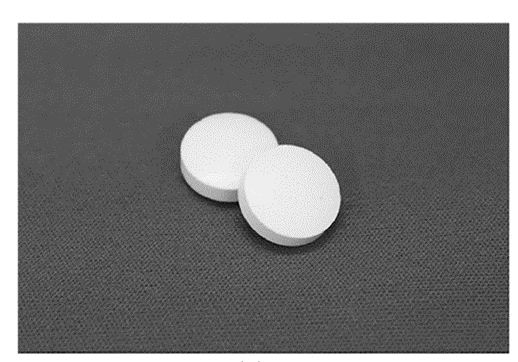


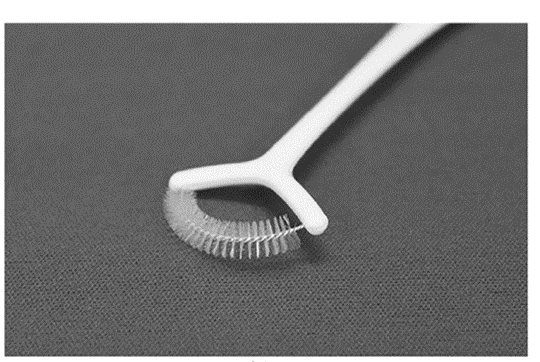


Supplemental Figure 1. (A) Tongue brush, (B) Oral care tablet.

Supplement: Supplementary file 1 — Supporting information. [file CRE2-8-721-s001.docx]
