# Supplementary material for: Oral care tablet containing kiwifruit powder affects tongue coating microbiome
Source: Clin Exp Dent Res. 2022 May 17;8(3):721–8. doi: 10.1002/cre2.591 (PMC9209808; doi:10.1002/cre2.591)
Supplement: Supplementary file 2 — Supporting information. [file CRE2-8-721-s002.docx]

Supplemental Figure 1 (A) Tongue brush, (B) Oral care tablet.

| Supplemental Table 1. Composition of oral care tablet |
| --- |
| Reduced palatinose |
| Erythritol |
| Malted rice extract powder |
| Kiwifruit powder |
| Sorbitol |
| Food flavor |
| Acidulant |
| Emulsifier |
| Silica particulate |
| Calcium Stearate |
| Sweetener |
| Food color |
| Tea extract |
